# Supplementary material for: Manipulation of Alcohol and Short-Chain Fatty Acids in the Metabolome of Commensal and Virulent Klebsiella pneumoniae by Linolenic Acid
Source: Microorganisms. 2020 May 21;8(5):773. doi: 10.3390/microorganisms8050773 (PMC7285277; doi:10.3390/microorganisms8050773)
Supplement: Supplementary file 1 [file microorganisms-08-00773-s001.zip › 05172020 Microorganisms Supplmentary second resubmission.pdf]

Supplementary Material

Brief Report

Manipulation of Alcohol and Short-chain Fatty Acids in the Metabolome of Commensal and Virulent *Klebsiella pneumoniae* by Linolenic Acid

Ryan Yuki Huang[1, 2], Deron Raymond Herr[3], and Shabbir Moochhala[3*]

[1] Canyon Crest Academy, San Diego, 92130, CA, USA.
[2] Department of Mechanical and Aerospace Engineering, University of California, San Diego, 92093, CA, USA.
[3] Department of Pharmacology, National University of Singapore, 117600, Singapore.

[*]**Reprint requests to:** Dr. Shabbir Moochhala, National University of Singapore, Singapore.

E-mail: E-mail: phcsmm@nus.edu.sg

Tel: +65-8511-0112

AGATGCGCAGCTACACATGCAGTCGAGCGGTAGCACAGAGAGCTTGCTCTCGGGTGACGAGCGG
CGGACGGGTGAGTAATGTCTGGGAAACTGCCTGATGGAGGGGGATAACTACTGGAAACGGTAGC
TAATACCGCATAATGTCGCAAGACCAAAGTGGGGGACCTTCGGGCCTCATGCCATCAGATGTGC
CCAGATGGGATTAGCTAGTAGGTGGGGTAATGGCTCACCTAGGCGACGATCCCTAGCTGGTCTGA
GAGGATGACCAGCCACACTGGAACTGAGACACGGTCCAGACTCCTACGGGAGGCAGCAGTGGG
GAATATTGCACAATGGGCGCAAGCCTGATGCAGCCATGCCGCGTGTGTGAAGAAGGCCTTCGGG
TTGTAAAGCACTTTCAGCGGGGAGGAAGGCGATAAGGTTAATAACCTTGTCGATTGACGTTACCC
GCAGAAGAAGCACCGGCTAACTCCGTGCCAGCAGGGCCGGGTAATAGA

Figure S1. 16S ribosomal RNA (rRNA) sequence of human isolates of *K. pneumoniae* using the 16S rRNA forward (F) 5'-GAG TTT GAT CCT GGC TCA-3′ and reverse (R) 5'-ACG GCT AAC TTG TTA CGA CT-3′ primers.
